# High-Sensitivity C-Reactive Protein and Acute Kidney Injury in Patients with Acute Myocardial Infarction: A Prospective Observational Study

**DOI:** 10.3390/jcm8122192

**Published:** 2019-12-12

**Authors:** Nicola Cosentino, Stefano Genovese, Jeness Campodonico, Alice Bonomi, Claudia Lucci, Valentina Milazzo, Marco Moltrasio, Maria Luisa Biondi, Daniela Riggio, Fabrizio Veglia, Roberto Ceriani, Katia Celentano, Monica De Metrio, Mara Rubino, Antonio L. Bartorelli, Giancarlo Marenzi

**Affiliations:** 1 Centro Cardiologico Monzino IRCCS, 20138 Milan, Italy; nicola.cosentino@ccfm.it (N.C.); stefano.genovese@ccfm.it (S.G.); jeness.campodonico@ccfm.it (J.C.); alice.bonomi@ccfm.it (A.B.); claudia.lucci87@gmail.com (C.L.); valentina.milazzo@ccfm.it (V.M.); marco.moltrasio@ccfm.it (M.M.); maria.biondi@ccfm.it (M.L.B.); daniela.riggio@ccfm.it (D.R.); fabrizio.veglia@ccfm.it (F.V.); roberto.ceriani@ccfm.it (R.C.); katia.celentano@ccfm.it (K.C.); monica.demetrio@ccfm.it (M.D.M.); mara.rubino@ccfm.it (M.R.); antonio.bartorelli@ccfm.it (A.L.B.); 2 Department of Biomedical and Clinical Sciences “Luigi Sacco”, University of Milan, 20138 Milan, Italy

**Keywords:** high-sensitivity C-reactive protein, acute kidney injury, acute myocardial infarction, in-hospital prognosis

## Abstract

**Background.** Accumulating evidence suggests that inflammation plays a key role in acute kidney injury (AKI) pathogenesis. We explored the relationship between high-sensitivity C-reactive protein (hs-CRP) and AKI in acute myocardial infarction (AMI). **Methods.** We prospectively included 2,063 AMI patients in whom hs-CRP was measured at admission. AKI incidence and a clinical composite of in-hospital death, cardiogenic shock, and acute pulmonary edema were the study endpoints. **Results**. Two-hundred-thirty-four (11%) patients developed AKI. hs-CRP levels were higher in AKI patients (45 ± 87 vs. 16 ± 41 mg/L; *p* < 0.0001). The incidence and severity of AKI, as well as the rate of the composite endpoint, increased in parallel with hs-CRP quartiles (*p* for trend <0.0001 for all comparisons). A significant correlation was found between hs-CRP and the maximal increase of serum creatinine (R = 0.23; *p* < 0.0001). The AUC of hs-CRP for AKI prediction was 0.69 (*p* < 0.001). At reclassification analysis, addition of hs-CRP allowed to properly reclassify 14% of patients when added to creatinine and 8% of patients when added to a clinical model. **Conclusions.** In AMI, admission hs-CRP is closely associated with AKI development and severity, and with in-hospital outcomes. Future research should focus on whether prophylactic renal strategies in patients with high hs-CRP might prevent AKI and improve outcome.

## 1. Introduction

Acute kidney injury (AKI) is a frequent complication of acute myocardial infarction (AMI), and is associated with an increased risk of prolonged hospital stay, cardiovascular events, renal failure, and mortality [1,2]. The occurrence of AKI in AMI is a multifactorial event that is promoted by underlying renal dysfunction, but it is often influenced by multiple contributing factors, such as hemodynamic impairment and use of contrast agents [3,4]. An early risk stratification for AKI is important because it might identify high-risk patients who could benefit from preventive measures, such as adequate hydration and limitation of contrast dose [5,6].

Acute kidney injury is a complex disorder with a wide variety of etiologies and corresponding risk factors. The common causes for AKI include renal ischemia/reperfusion injury, systemic inflammatory processes/sepsis, hemodynamic impairment, and nephrotoxicity [7]. Among them, inflammation plays a critical role, and several experimental studies have demonstrated an important contribution of intra-renal and systemic inflammation in promoting parenchymal injury, repair, and fibrosis of the kidney [8,9,10]. Growing attention has been focused on C-reactive protein (CRP), a simply detectable inflammation biomarker, as a possible predictor of AKI and it has been recently recognized that CRP actively contributes in the pathogenesis and progression of AKI, by exacerbating local inflammation, impairing the proliferation of damaged tubular epithelial cells, and promoting the fibrosis of injured renal tissue [11,12,13,14,15]. Moreover, physicians have now become accustomed to use high-sensitivity CRP (hs-CRP), when considering vascular disease risk stratification, as opposed to the use of standard CRP assays that monitor infections and other inflammatory conditions [16,17,18]. In particular, to assess the cardiovascular risk, CRP should be measured by highly sensitive methods that are capable of reliably measuring concentrations within the healthy reference interval [16,17,18].

Emerging evidence showed that serum level of CRP acts as a risk factor and, at the same time, as a potential causal factor for both AKI development and severity, which is also seen in AMI patients [15]. In coronary artery disease patients, Gao et al. [19], who were the first to perform a retrospective analysis on 4522 patients undergoing elective percutaneous coronary intervention (PCI) with drug-eluting stents, found that elevated pre-procedural CRP was associated with a progressively increased risk of contrast-induced AKI. Similarly, a recent retrospective analysis by Han et al. [20], including 1656 patients undergoing coronary artery bypass grafting, showed that pre-operative CRP level is a predictor of post-operative AKI. Finally, Shacham et al. prospectively investigated 562 STEMI patients undergoing primary PCI, and they reported that those with hs-CRP levels >9 mg/L at hospital admission had higher AKI (17% vs. 6%) and 30-day mortality (11% vs. 1%) rates than patients with hs-CRP levels below this limit [21]. Thus, it could be hypothesized that, in AMI patients, the association between high levels of hs-CRP and worse in-hospital prognosis is mediated, at least in part, by AKI occurrence.

In the present study, we prospectively investigated whether hs-CRP levels measured at hospital admission might predict AKI, as well as its severity, in a large consecutive cohort of AMI patients. Moreover, we analyzed the possible causal relationship between hs-CRP, AKI, and in-hospital clinical outcomes.

## 2. Material and Methods

**Study Population**. This prospective observational study was conducted at the Centro Cardiologico Monzino between 1 June 2010 and 31 May 2019. We enrolled all consecutive AMI patients (both STEMI and non-ST elevation myocardial infarction [NSTEMI] patients) admitted to the Intensive Cardiac Care Unit (ICCU). We excluded patients in chronic peritoneal or hemodialysis treatment (*n* = 2), those with concomitant systemic inflammatory conditions (*n* = 135) (including active infection [*n* = 117]) or malignancies (*n* = 38), and those who died or underwent emergency cardiac surgery before at least two consecutive serum creatinine (sCr) values had been collected (*n* = 34). The Ethics Committee of our Institute (Centro Cardiologico Monzino) approved the study (study protocol number R520-CCM549), and written informed consent was obtained from all patients.

**Study Protocol.** In all patients, hs-CRP was measured at hospital admission through a Cobas^®^ assay (particle-enhanced immunoturbidimetric assay) on Cobas c501 (Roche) [22]. Serum creatinine was also measured at hospital admission and daily during ICCU stay. Glomerular filtration rate (eGFR) was estimated using the Modification of Diet in Renal Disease Study (MDRD) equation [23]. Acute kidney injury was defined applying the Acute Kidney Injury Network classification, according to the maximum sCr increase recorded between baseline (hospital admission) and the first 72 h [24]. In particular, Stage 1 AKI was defined as a ≥ 0.3 mg/dL sCr increase; Stage 2 as a >2- to 3-fold sCr increase; Stage 3 as a >3-fold sCr increase or sCr ≥ 4.0 mg/dL with an acute increase >0.5 mg/dL, or need for renal replacement therapy (RRT), irrespective of the stage at the time of RRT.

Study patients received standard medical treatment and coronary revascularization (primary or early PCI in most cases) according to the current standards of care recommended by published guidelines. Nonionic, low-osmolality contrast agents were used in all patients. In NSTEMI patients, isotonic (0.9%) saline was administered intravenously at a rate of 1 mL/kg/h for 12 h, before and after contrast exposure. In STEMI patients, hydration was given for 12 h after PCI. In patients with left ventricular ejection fraction (LVEF) <40%, or those who showed overt heart failure, the hydration rate was reduced to 0.5 mL/kg/h.

The primary outcome for this study was the incidence of AKI. A composite of in-hospital mortality, cardiogenic shock, and acute pulmonary edema was also evaluated as a secondary clinical endpoint of the study. We used this combined endpoint because cardiogenic shock and acute pulmonary edema are the complications most closely associated with mortality in AMI, and they are the clinical manifestations of acute ventricular dysfunction, which is known to be associated with conditions of acute inflammation [25,26]. Cardiogenic shock was defined as persistent systolic arterial pressure ≤80 mmHg, with evidence of vital organ hypoperfusion caused by severe left ventricular dysfunction, right ventricular infarction, or mechanical complications of infarction, and not due to hypovolemia, hemorrhage, bradyarrhythmias, or tachyarrhythmias. Acute pulmonary edema was defined as respiratory distress, tachypnea, and orthopnea with rales over the lung fields and arterial oxygen saturation <90%, despite high inspired oxygen concentration. To avoid interference, each patient could only account for one event classification.

**Statistical Analysis.** A sample size of 1,060 patients was calculated under the following assumptions: 10% overall incidence of AKI [2], with an expected 5% and 15% incidence in patients in the first and last hs-CRP quartiles, respectively. This sample size allowed a 90% statistical power in assessing a significant difference (α error of 0.0125 considering the Bonferroni correction for 4 comparisons) of the incidence of AKI between quartiles. This sample (*n* = 1060) also allowed a 70% statistical power when an overall incidence of 15% of the combined clinical endpoint was considered [27], with an expected 10% and 20% incidence in patients in the first and last hs-CRP quartiles, respectively. To increase the statistical power from 70% to 90% regarding the combined clinical endpoint, 2,000 patients were evaluated, and they represented the final sample size.

Continuous variables are presented as mean ± SD, and they were compared using the *t*-test for independent samples. Non-normally distributed variables are presented as median and interquartile ranges, and they were compared with the Wilcoxon rank-sum test. Categorical data were compared using the chi-square test or the Fisher exact test, as appropriate. Trends of AKI incidence across hs-CRP quartiles were assessed by Mantel–Haenszel chi-square.

The identification of independent predictors of AKI in the whole population was assessed by a logistic regression analysis with the initial set of potential predictors, including variables, found to be significantly (*p* < 0.05) associated with AKI, during univariate analysis. Results are presented as odds ratio (OR) with a 95% confidence intervals (CI).

The receiver operating characteristic (ROC) curves were calculated to measure the ability of the considered variables to predict AKI. The results are presented as area under the curve (AUC) with 95% CI. AUC were compared as recommended by DeLong et al. [28]. Net reclassification improvement (NRI) was used to identify the possible additional prognostic value of hs-CRP when added to admission sCr and to the variables found to be independently associated with AKI, during multivariate analysis (age, sCr, LVEF, admission glycemia, and AMI type [STEMI vs. NSTEMI]).

Path analysis using structural equation modeling was also performed in order to explore the paths through which hs-CRP affects the endpoints [29]. The results of the path analysis are presented as standardized regression coefficients (β).

All tests were two-tailed, and *p* < 0.05 was required for statistical significance. All analyses were performed using SAS version 9.4 (SAS Institute, Cary, NC, USA). Reclassification statistics were assessed with the SAS macros published by Cook and Ridker [30].

## 3. Results

A total of 2,272 AMI patients were initially enrolled in this study. After screening for eligibility, 2,063 AMI patients (mean age 67 ± 12 years, 73% men, 49% STEMI patients) were included in the final analysis. Of them, 234 (11%) developed AKI (13% of STEMI and 10% of NSTEMI patients; *p* = 0.05). Stage 1 AKI occurred in 173 (8%) patients, and Stage 2–3 AKI occurred in 61 (3%) patients. Thirty-two AKI patients (2% of the entire population) required RRT.

The baseline clinical characteristics of the patients with and without AKI are shown in Table 1. Patients with AKI were older and more likely to have co-morbidities, prior cardiovascular events, and lower eGFR and LVEF. Moreover, AKI patients experienced a higher rate of in-hospital complications, including mortality (Table 1).

**Figure 1:**
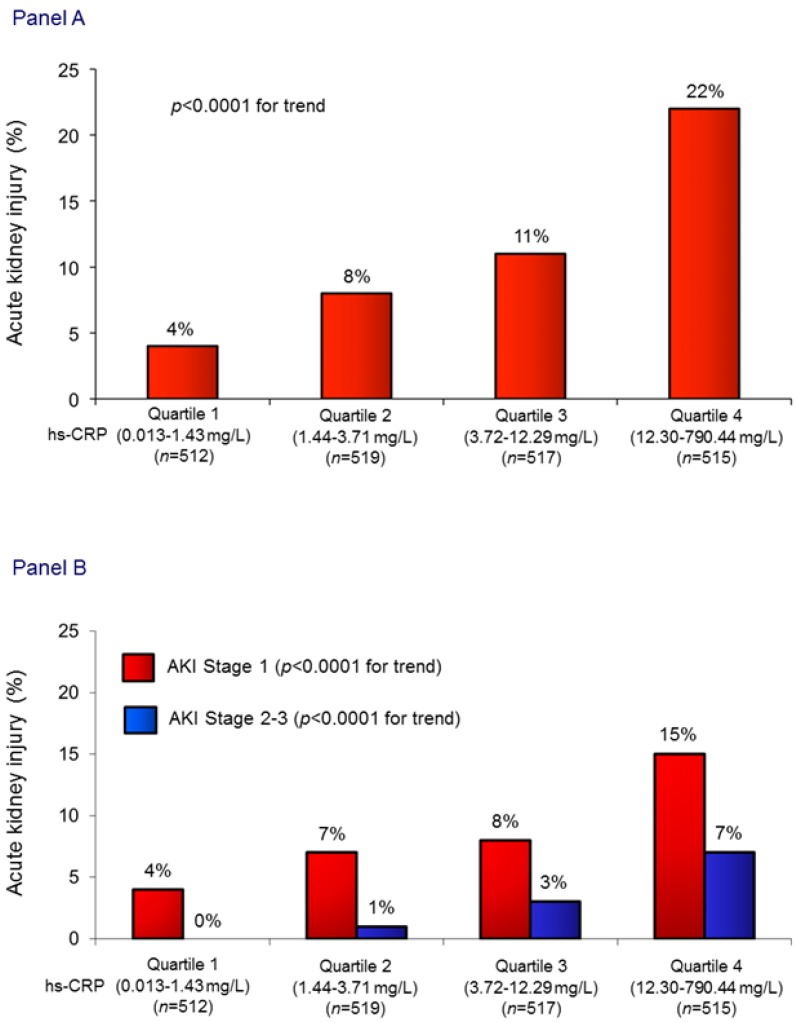
(**A**) Acute kidney injury (AKI) rates in the study patients, grouped according to admission high-sensitivity C-reactive protein (hs-CRP) quartiles. (**B**) Acute kidney injury rates stratified according to its severity in hs-CRP quartiles.

The mean admission hs-CRP value in the entire population was 19 ± 49 mg/L. Patients who developed AKI showed markedly higher hs-CRP values than those without AKI (45 ± 87 vs. 16 ± 41 mg/L; *p* < 0.0001). The incidence of AKI, as well as its severity, increased in parallel with hs-CRP quartiles (Figure 1). Notably, also when a cut-off value of sCr increase ≥0.5 mg/dL was considered, AKI incidence significantly increased in parallel with hs-CRP quartiles (3%, 9%, 11%, and 18%, respectively; *p* for trend <0.0001).

**Figure 2:**
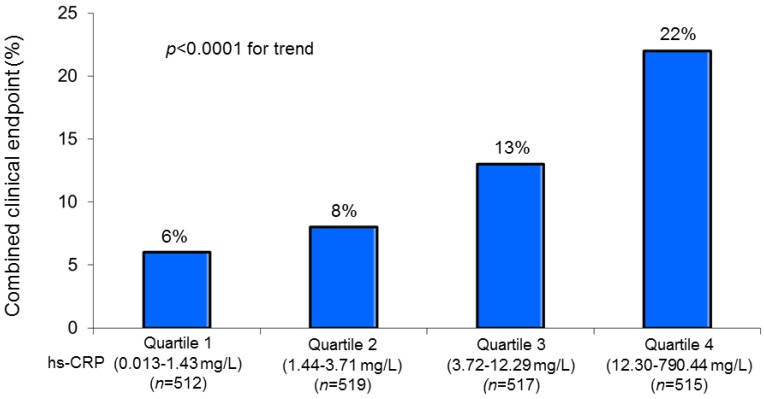
Combined clinical endpoint (in-hospital mortality, acute pulmonary edema, and cardiogenic shock) rate in the study patients grouped according to the admission high-sensitivity C-reactive protein (hs-CRP) quartiles.

A similar relationship with hs-CRP quartiles was also observed for the incidence of the combined clinical endpoint (Figure 2).

Moreover, a significant correlation was found between admission hs-CRP values and the subsequent maximal increase of sCr (R = 0.23; *p* < 0.0001).

**Figure 3:**
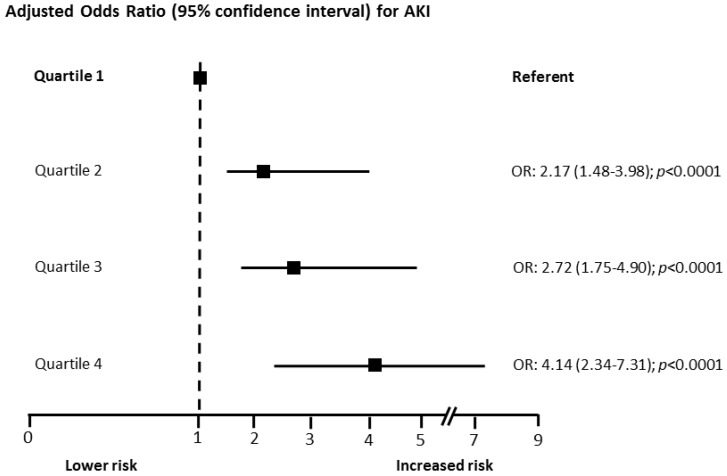
Adjusted odds ratios (OR) and 95% confidence intervals for acute kidney injury (AKI) risk according to high-sensitivity C-reactive protein quartiles. Odd ratios were adjusted for baseline clinical variables found to be independently associated with AKI during multivariate analysis (age, serum creatinine, left ventricular ejection fraction, admission glycemia, and acute myocardial infarction type).

Figure 3 shows the OR for AKI, according to hs-CRP quartiles adjusted for baseline clinical variables that were found to be independently associated with AKI (age, sCr, LVEF, admission glycemia, and AMI type).

In the whole population, when hs-CRP was evaluated in terms of its capacity to predict AKI, the AUC was 0.69 (95% CI 0.65 to 0.73; *p* < 0.001). Notably, hs-CRP showed a similar capacity to predict AKI when STEMI (AUC 0.69; 95% CI 0.64 to 0.74; *p* < 0.001) and NSTEMI (AUC 0.69; 95% CI 0.63 to 0.74; *p* < 0.001) patients were considered separately. High-sensitivity C-reactive protein significantly increased the accuracy to predict AKI when added to admission sCr and to the variables found to independently predict AKI during multivariate analysis. During the reclassification analysis, the addition of hs-CRP to admission sCr allowed to properly reclassify 14% of the patients. Similarly, when hs-CRP was added to the independent clinical variables identified in our cohort, 8% of the patients were properly reclassified (Table 2). 

**Figure 4:**
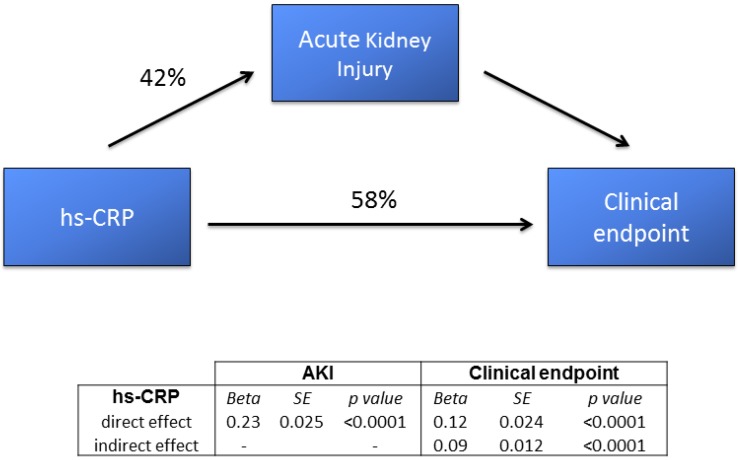
Sequential path analysis diagram depicting the interrelationships (direct and indirect effects) between admission high-sensitivity C-reactive protein (hs-CRP), acute kidney injury (AKI), and the combined clinical endpoint (in-hospital mortality, acute pulmonary edema, and cardiogenic shock). SE—standard error.

The results of the path analysis with the estimated direct and indirect effects of admission hs-CRP on AKI and primary endpoint occurrence are presented in Figure 4.

High-sensitivity-CRP was significantly associated with both variables of interest. In particular, it was associated with the clinical endpoint through both a direct effect (58% of its overall effect) and an indirect effect (42% of its overall effect), which passed through the development of AKI.

## 4. Discussion

The major finding of the study was that hs-CRP levels measured at hospital admission in patients with AMI were independently associated with AKI risk, its severity, and in-hospital clinical outcomes.

The development of AKI in AMI patients was strongly associated with a worse prognosis. Increased in-hospital morbidity and mortality and prolonged hospital stay were reported, along with a higher risk of progression to end-stage renal disease and subsequent hospitalization for cardiovascular and renal events [1,2,31]. Thus, AKI prediction is a clinical priority to identify high-risk AMI patients, in whom preventive strategies should be implemented in order to minimize the risk of AKI and improve the outcome.

In our study, we prospectively evaluated hs-CRP at hospital admission as a predictor of AKI in a large consecutive cohort of AMI patients, including both STEMI and NSTEMI. Similar to other reports [1,2,3,4], 11% of our study patients developed AKI. Its occurrence was associated with an in-hospital mortality strikingly higher than that observed in patients without AKI. Of note, hs-CRP levels were also significantly higher in patients with AKI than in those without, and it increased in parallel with increasing AKI severity. At the same time, the risk of AKI increased in parallel with admission hs-CRP values, while a significant relationship between hs-CRP value and maximal sCr increase was observed in the entire study population. Remarkably, risk prediction accuracy improved after the addition of hs-CRP to admission sCr, the most common predictor of AKI in AMI studies, as well as in studies performed in other clinical settings [32,33,34]. The accuracy of AKI prediction also improved when hs-CRP was added to a clinical model including other well-known independent predictors of AKI in AMI (age, LVEF, sCr, admission glycemia, and AMI type) [33], allowing a reclassification of AKI risk in nearly 10% of patients.

Given the recognized close association between hs-CRP and in-hospital outcome in AMI [17], we hypothesized that inflammation might directly contribute to acute organ injury and, in turn, affect patient prognosis. Indeed, higher admission levels of hs-CRP in AMI have been reported to predict left ventricular dysfunction [35] and heart failure [36], suggesting that inflammatory processes play an independent role in cardiac function impairment during AMI. To better elucidate this point, in addition to AKI, we considered a combined clinical endpoint including acute pulmonary edema, cardiogenic shock, and death, which are clinical equivalents of acute ventricular dysfunction. Our findings seem to confirm such a hypothesis, suggesting that high levels of hs-CRP are closely associated with an increased risk of AKI and, at the same time, with a worse in-hospital prognosis. This is further supported by the results of the path analysis, demonstrating that the association between hs-CRP and in-hospital outcome is the result of both a direct effect (58% of the hs-CRP overall effect) and an indirect effect, which passes through the development of AKI (42% of its overall effect).

The mechanisms underlying the association between hs-CRP and AKI risk remain uncertain and they require further exploration. However, based on our results and on those of the existing literature, there is compelling evidence that elevation of hs-CRP level in AMI patients might not just be a marker of inflammation, it might also directly contribute to the inflammatory state causing injury to different organs, and particularly to kidney and heart, whose dysfunctions are the most important variables associated with AMI prognosis [37,38,39]. Indeed, hs-CRP has been demonstrated to reduce nitric oxide production and to impair antioxidant defenses, resulting in endothelial dysfunction and decreased activity of renal vasodilators, both major contributors to AKI development [11,14,40]. At the same time, in conditions characterized by acute and systemic inflammation, such as severe burn, trauma, or sepsis, a decrease in cardiac myocyte contractility often occurs. The mechanisms of myocardial dysfunction are related with the inflammatory response mediated by cytokines, oxidative stress, endothelial dysfunction, and mitochondrial abnormalities. In particular, cytokines act as the most important mediators of the inflammatory process. Tumor necrosis factor-α, interleukin-1β, and interleukin-6 are increased during an inflammatory response, and they have been shown to have a negative inotropic effect on cardiomyocyte contractility and, at the same time, to cause acute renal dysfunction [25,26].

Our findings might have some relevant clinical implications. Previous meta-analyses and studies demonstrated that the administration of statins significantly reduces AKI incidence, as well as myocardial infarct size, in AMI patients [41,42]. As statins are known to have pleiotropic and anti-inflammatory effects, the early administration of high-dose statins might blunt the acute inflammatory response in AMI and exert renal-protective and cardio-protective effects. Moreover, hs-CRP measurement in the first hours of hospital admission could be utilized easily to identify high-risk AMI patients in whom early prophylactic strategies aimed at preventing AKI or at reducing its severity, like furosemide-induced diuresis with matched hydration, should be implemented [6].

The strengths of our study include a well-characterized AMI population, the adjustment for several risk factors, a special focus on the relationship between hs-CRP and AKI, and the use of an innovative statistical methodology (path analysis) that is able to elucidate the possible causal pathways between hs-CRP and the outcomes of interest. However, some limitations of the study should be mentioned. First, we included a population admitted to a single center. Second, sCr at hospital admission may not represent a true baseline renal function in AMI patients because AKI could have already occurred due to hemodynamic impairment, potentially underestimating AKI incidence and severity. Thus, our data cannot ascertain whether hs-CRP is a predictor or an early marker of AKI. Nevertheless, our path analysis might suggest a causal link between admission hs-CRP and AKI. Third, we did not serially evaluate hs-CRP in our patients. Notably, it has been recently reported that changes in hs-CRP over time, rather than a single value measured at hospital admission, are more accurate predictors of AKI in STEMI patients undergoing primary PCI [43]. It is also possible that other inflammation indices that we have not considered, such as the CRP to albumin ratio, might be more accurate in predicting AKI in AMI patients [44]. Finally, clinical events were assessed during hospital stay only, precluding to draw any inference from our data regarding long-term outcomes.

## 5. Conclusions

In conclusion, in patients hospitalized with AMI, hs-CRP measured at admission is closely associated with AKI development and severity. Future research should focus on whether targeted prophylactic renal strategies in high-risk patients, selected according to hs-CRP value, might prevent AKI and improve clinical outcome.

## Figures and Tables

**Table 1:** Baseline characteristics and in-hospital complications of the study patients according to the occurrence of acute kidney injury.

Variable	Acute Kidney Injury		
	No	Yes	*p* Value
(*n* = 1829)	(*n* = 234)		
Age (years)	66 ± 12	74 ± 11	<0.0001
Male sex, *n* (%)	1351 (74%)	165 (71%)	0.27
Body weight (kg)	76 ± 14	76 ± 16	0.88
Diabetes mellitus, *n* (%)	386 (21%)	87 (37%)	<0.0001
Hypertension, *n* (%)	1157 (63%)	178 (76%)	0.0001
Smokers, *n* (%)	1003 (55%)	95 (41%)	<0.0001
Hyperlipidemia, *n* (%)	900 (49%)	129 (55%)	0.09
Prior myocardial infarction, *n* (%)	451 (25%)	82 (35%)	0.0006
Prior CABG, *n* (%)	210 (11%)	41 (18%)	0.007
Prior PCI, *n* (%)	450 (25%)	66 (28%)	0.22
Left ventricular ejection fraction (%)	51 ± 11	41 ± 14	<0.0001
STEMI, *n* (%)	885 (48%)	131 (56%)	0.03
CA/PCI during hospitalization, *n* (%)	1724 (94%)	206 (88%)	0.0006
**Laboratory values at hospital admission**			
Serum creatinine (mg/dL)	1.0 ± 0.4	1.4 ± 0.9	<0.0001
eGFR (ml/min/1.73m^2^)	80 ± 26	63 ± 30	<0.0001
Hemoglobin (g/dL)	13.7 ± 1.8	13.1 ± 2.1	<0.0001
Blood glucose (mg/dL)	146 ± 57	192 ± 88	<0.0001
hs-TnI (ng/L)	5223 ± 20,634	12,992 ± 50,065	<0.0001
**Medication before hospital admission**			
Aspirin, *n* (%)	642 (35%)	108 (46%)	0.0009
Statins, *n* (%)	601 (33%)	90 (38%)	0.11
Beta-blockers, *n* (%)	632 (28%)	100 (24%)	0.01
ACE/AR blockers, *n* (%)	718 (35%)	93 (40%)	0.89
Oral anticoagulants, *n* (%)	91 (5%)	17 (7%)	0.29
**In-hospital complications**			
In-hospital death, *n* (%)	9 (0.5%)	30 (13%)	<0.0001
Cardiogenic shock, *n* (%)	58 (3%)	59 (25%)	<0.0001
Acute pulmonary edema, *n* (%)	115 (6%)	99 (42%)	<0.0001
Combined clinical endpoint, *n* (%)	141 (8%)	119 (51%)	<0.0001
Mechanical ventilation, *n* (%)	32 (2%)	46 (20%)	<0.0001
Atrial fibrillation, *n* (%)	147 (8%)	62 (26%)	<0.0001
VT/VF, *n* (%)	127 (7%)	41 (18%)	<0.0001
High-degree CD, *n* (%)	60 (3%)	14 (6%)	0.04
Major bleeding, *n* (%)	41 (2%)	31 (13%)	<0.0001
ICCU length of stay (days) *	4 (3–4)	5 (4–8)	<0.001 †

ACE—angiotensin-converting enzyme; AR—angiotensin II receptor; CA—coronary angiography; CABG—coronary artery bypass graft; CD—conduction disturbances; eGFR—estimated glomerular filtration rate; hs-TnI—high-sensitivity troponin I; ICCU—intensive cardiac care unit; PCI—percutaneous coronary intervention; STEMI—ST-segment elevation myocardial infarction; VF—ventricular fibrillation; and VT—ventricular tachycardia. * Median and interquartile range. † By non-parametric Wilcoxon rank-sum test.

**Table 2:** Area under the curve of admission high-sensitivity C reactive protein (hs-CRP) added to serum creatinine (sCr) and to the clinical variables found to be independently associated with acute kidney injury, for the prediction of the primary endpoint (acute kidney injury), and reclassification statistics comparisons.

	AUC (95% CI)	*p* Value AUC	*p* Value for AUC Comparison	NRI (95% CI)	*p* Value NRI
Admission sCr	0.66 (0.62–0.70)	<0.001	-	-	-
Admission hs-CRP + sCr	0.72 (0.68–0.76)	<0.001	<0.001	14% (10–17)	0.01
Clinical predictors *	0.79 (0.77–0.83)	<0.001	-	-	-
Admission hs-CRP + clinical predictors *	0.81 (0.78–0.84)	<0.001	0.002	8% (3–12)	<0.001

AUC—area under the curve; CI—confidence intervals; NRI—net reclassification improvement. *Age, admission serum creatinine, left ventricular ejection fraction, admission glycemia, and acute myocardial infarction type.
